# Vegetation as self‐adaptive coastal protection: Reduction of current velocity and morphologic plasticity of a brackish marsh pioneer

**DOI:** 10.1002/ece3.1904

**Published:** 2016-02-12

**Authors:** Jana Carus, Maike Paul, Boris Schröder

**Affiliations:** ^1^ Environmental Systems AnalysisInstitute of GeoecologyTechnische Universität BraunschweigLanger Kamp 19c38106BraunschweigGermany; ^2^ Institute of Earth and Environmental SciencesUniversity of PotsdamKarl‐Liebknecht‐Str. 24‐2514476PotsdamGermany; ^3^ Forschungszentrum KüsteMerkurstr. 1130419HannoverGermany; ^4^ Berlin‐Brandenburg Institute of Advanced Biodiversity Research (BBIB)BerlinGermany

**Keywords:** Adaptive value, *Bolboschoenus maritimus*, brackish marsh, flow velocity, mechanical pressure, morphological adaptation, phenotypic plasticity, pioneer zone

## Abstract

By reducing current velocity, tidal marsh vegetation can diminish storm surges and storm waves. Conversely, currents often exert high mechanical stresses onto the plants and hence affect vegetation structure and plant characteristics. In our study, we aim at analysing this interaction from both angles. On the one hand, we quantify the reduction of current velocity by *Bolboschoenus maritimus,* and on the other hand, we identify functional traits of *B. maritimus’* ramets along environmental gradients. Our results show that tidal marsh vegetation is able to buffer a large proportion of the flow velocity at currents under normal conditions. Cross‐shore current velocity decreased with distance from the marsh edge and was reduced by more than 50% after 15 m of vegetation. We were furthermore able to show that plants growing at the marsh edge had a significantly larger diameter than plants from inside the vegetation. We found a positive correlation between plant thickness and cross‐shore current which could provide an adaptive value in habitats with high mechanical stress. With the adapted morphology of plants growing at the highly exposed marsh edge, the entire vegetation belt is able to better resist the mechanical stress of high current velocities. This self‐adaptive effect thus increases the ability of *B. maritimus* to grow and persist in the pioneer zone and may hence better contribute to ecosystem‐based coastal protection by reducing current velocity.

## Introduction

Tidal marshes play an important role for coastal flood defence (Kirwan and Megonigal 2013; Temmerman et al. 2013). By reducing current velocity and attenuating waves (Christiansen et al. 2000; Temmerman et al. 2005), marsh vegetation diminishes the destructive effects of storm surges and storm waves (Gedan et al. 2010; Shepard et al. 2011). Moreover, it reduces shoreline erosion and increases sedimentation (Neumeier and Ciavola 2004). An intact marsh vegetation belt hereby promotes natural accretion of sediments and has the ability to adapt to projected sea level changes (Kirwan et al. 2010). Temmerman et al. (2013) argued that the maintenance of conventional coastal engineering solutions may become unsustainable with increasing flood risk propelled by rising sea levels and therefore, the creation or restoration of coastal ecosystems could replace or improve and support conventional levee structures. For the plants, however, the costs of living in such a stressful environment are high. In tidal marshes, vegetation is flooded up to twice a day, often for several hours and waves and currents often exert high mechanical stress on the plants (Coops et al. 1994; Bal et al. 2011). As vegetation buffers current and wave energy to some extent, the hydrodynamic forces together with related mechanical stresses are not evenly distributed in the vegetation belt. Thus, vegetation structure and zonation are influenced by hydrodynamic forcing. Because current often has an adaptive effect on growth traits (Puijalon and Bornette 2004; Puijalon et al. 2005; Szmeja and Galka 2008), even ramets of one species can vary within the vegetation belt (Barrett et al. 1993) and thereby enhance the ability of vegetation to serve as self‐adaptive flood defence. Hence, hydrodynamic forcing influences vegetation structure and zonation, as well as variability of individuals in populations (Barrett et al. 1993).

### Effect of vegetation on current velocity

In tidally influenced estuaries, currents are strongly affected by the rise and fall of the water level (Le Hir et al. 2000). In general, these currents can be split into two components: a long‐shore component, which is shore‐parallel, and a cross‐shore component, which runs vertically to the shore (Le Hir et al. 2000). In these systems, long‐shore current velocity is mainly generated by water draining into the sea and by the inflow of the rising tide. The highest velocities are reached during storms coinciding with spring tides. In contrast, cross‐shore currents result from filling and emptying of the intertidal flats. Cross‐shore current velocity depends mainly on the tidal range (McAnally and Mehta 2001) and the width of the mudflat (Le Hir et al. 2000). It can exceed long‐shore current velocity when the intertidal flat is particularly wide, and/or when long‐shore currents are reduced by the presence of natural or man‐made obstructions.

Previous studies found that the presence of plants strongly reduces current velocity inside the vegetation (Neumeier and Ciavola 2004). The degree of reduction depends on the amount of dampening plant mass, thus on vegetation type, vegetation density, canopy height, and width of the vegetation belt (Leonard and Luther 1995; Christiansen et al. 2000; Neumeier and Ciavola 2004).

The reduction of current energy by vegetation has mostly been studied in laboratory flumes. These studies have enlarged knowledge on drag and turbulence caused by vegetation and its different effects on vertical flow and turbulence profiles (e.g. Nepf 1999; Nepf and Vivoni 2000; Temmerman et al. 2005). However, important differences exist between natural marshes and their laboratory models (Neumeier and Ciavola 2004). For example, the canopy used in flumes is much less complex than a naturally grown vegetation belt. Field studies published so far accounted for this complexity but flow velocity measurements did not explicitly consider the effect of vegetation by comparing measurements with and without living aboveground biomass. Several marsh species have been investigated, but to our knowledge no study explicitly considers *Bolboschoenus maritimus (L.) Palla* and its role in the attenuation of hydrodynamic forces, although it is one of the most common primary colonizers on brackish tidal flats (Boaden and Seed 1988).

### Morphological plant response

For many plant species it has been found that individuals differ in phenotype (Richards et al. 2005). These differences can, for example, occur through the creation of different phenotypes from one genotype as an adaptation to differing environmental conditions (Clausen et al. 1948; Richards et al. 2005). This phenotypic/morphologic plasticity can be very advantageous in spatially or temporally heterogeneous environments (Alpert and Simms 2002; Givnish 2002). For clonal plants, it has been hypothesized that phenotypic plasticity can result in the formation of specialized units and thus selective advantages in heterogeneous habitats (Eriksson and Jerling 1990; Alpert and Stuefer 1997).

The clonal plant *B. maritimus* forms populations consisting of many independent units, called ramets. These units are connected by rhizomes which (1) serve as storage organs (Suzuki and Stuefer 1999) and (2) facilitate vegetative dispersal (Karagatzides and Hutchinson 1991). For *B. maritimus* it has been shown that the proportion of aboveground dry matter increased at the expense of roots and rhizomes with increasing water depth (Clevering and Hundscheid 1998). Furthermore, the species is able to develop different groups of ramets specialized in sexual reproduction, resource storage, carbon assimilation, and vegetative growth depending on their position along the rhizome system (Lieffers and Shay 1981; Zákravský and Hroudová 1994). Charpentier and Stuefer (1999) showed that this specialization is affected by environmental conditions.

Species distribution and community dynamics of tidal marsh vegetation are highly affected by mechanical stress produced by hydrodynamic forces (Denny 1988; Vogel 1994). Especially in the pioneer zone, mechanical stress plays a dominant role in the establishment, survival, and expansion of vegetation (e.g. Bruno 2000; Houwing 2000; van Katwijk and Hermus 2000) because it can lead to breakage and uprooting of ramets. For submerged plants, it has been shown that current often has an adaptive effect on growth traits (Puijalon and Bornette 2004; Puijalon et al. 2005; Szmeja and Galka 2008) which can in some circumstances lead to greater hydrodynamic performance (Puijalon et al. 2005), i.e. the ability to withstand hydrodynamic forces induced by water movement. Hydrodynamic performance can be enhanced through alternative morphologies which either minimize mechanical forces (avoidance strategy, e.g. by adopting a streamlined form or by size reduction) or increase resistance to mechanical failure (tolerance strategy, e.g. by enhancing the proportion of strengthening tissue or by higher radial growth resulting in higher stem diameter) (Puijalon et al. 2008, 2011). The adaptive value of plant traits for withstanding mechanical forces can be assessed by stability measurements which determine bending stiffness and breaking force. Bending stiffness describes the resistance of a stem to bending. Breaking force is the maximal flexural force applied to the plant probe before it breaks. Although some studies approached phenotypic adaptation to environmental conditions (e.g. Clausen et al. 1948; Richards et al. 2005), to our knowledge, current velocity has so far not been addressed as influencing factor for growth characteristics of the tidal marsh pioneer *B. maritimus*.

As the effect of marsh vegetation on flow velocity is closely linked to its response to current energy (Butcher 1933), a holistic view is crucial for understanding the ability of vegetation to serve as self‐adaptive flood defence. Therefore, this study aims at analysing this interaction from both angles. On the one hand, we quantified the reduction of current velocity by *B. maritimus* by comparing field measurements with and without living vegetation as well as by estimating effect functions from the data. As the *B. maritimus* belt consists of very dense vegetation, we expected both long‐ and cross‐shore current velocity to be reduced directly behind the boundary between vegetation and open water. On the other hand, we identified the functional traits of *B. maritimus’* ramets which adapt to environmental conditions and assessed their adaptive value. As current velocity exerts high mechanical stress especially on ramets growing at the marsh edge, we anticipated some degree of morphological adaptation of these ramets.

To explore the possibility of the vegetation serving as self‐adaptive coastal protection, we measured current velocity with and without living vegetation, recorded ramet density and plant thickness during two growing periods at two locations in the Elbe estuary and assessed the plants adaptive value.

## Methods

### Study system and species description

**Figure 1:**
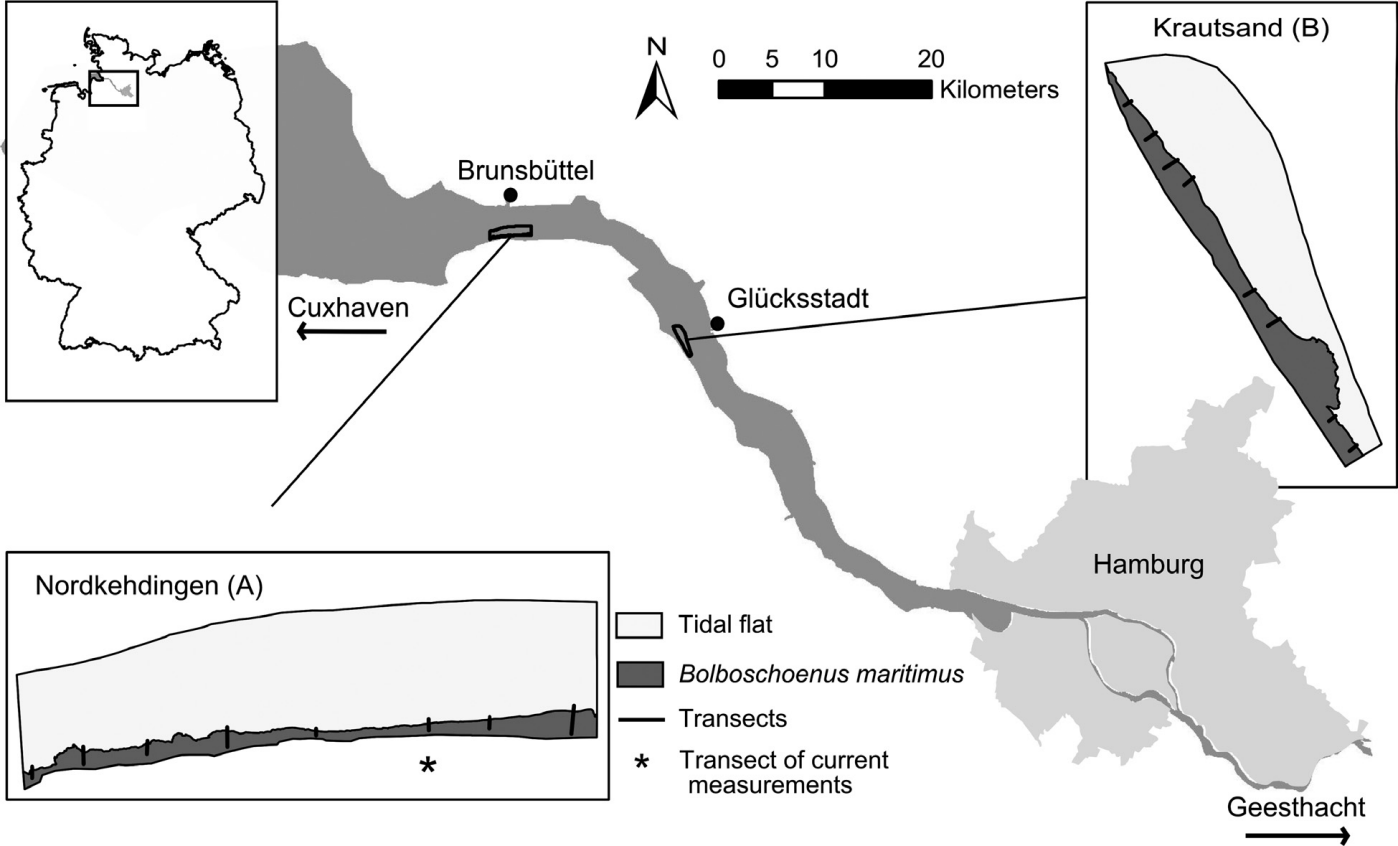
Location of the two study areas and position of the 16 transects in the Elbe estuary.

With a length of 170 km and a maximum width of 10 km, the estuary of the river Elbe is the largest estuary along the German coast. It is influenced by tides from the mouth in Cuxhaven to the weir in Geesthacht. The tidal range is highest in Hamburg (3.6 m) and decreases by 0.6 m in Cuxhaven (120 km downstream) and by 1.6 m in Geesthacht (40 km upstream). For the investigation of a tidally influenced marsh, it was crucial to select sites without dampening of the tidal influence (e.g. by embankments or wave breakers). For this reason, we selected one site in the nature reserve Nordkehdingen (A) (53°51′46.419″N, 9°5′50.027″E) and one site about 30 km upstream on the peninsula of Krautsand (B) (53°45′50.626″N, 9°22′46.052″E) (Fig. 1). The sites are both situated in the brackish part of the River Elbe and exhibit a mean soil water salinity of 4.5 ppt (A) and 1.5 ppt (B). The dominating species at both sites are *B. maritimus* at the waterfront and *Phragmites australis* further landwards. *B. maritimus* usually occurs in the pioneer zone of brackish marshes up to 1.2 m below mean high water and forms dense monospecific stands (Lieffers and Shay 1982a,b). It is a perennial clonal plant with a strongly branched system of rhizomes, interconnecting single ramets (Hroudová et al. 2007). In one growth period, *B. maritimus* can form many rhizomes and roots which contribute to a fast vegetative dispersal (Dykyjová 1986). In the study region, the above ground ramets sprout between March and April, grow up to 2 m high and consist of a triangular stem with up to 10 lineal leafs. Brown flowers rise above the leaves from June to August with oval spikelets clustered just below their tips. At the end of the growth period, all aboveground plant parts die back (Lillebø et al. 2003), and only belowground organs persist (Charpentier and Stuefer 1999). Due to the strong tidal influence, the littoral zone of the Elbe estuary is regularly flooded at high tide and drained at low tide. Maximum inundation height at the marsh edge ranges from 0.58 to 1.4 m in study area A and from 1.23 to 2.19 m in study area B. Maximum daily inundation time lies between 3.5 and 5.5 h in study area A and between 6.3 and 9.3 h in study area B. In most parts of the two study areas, *B. maritimus* is spreading with a dispersal rate of up to 9 m per year, but is retreating in other areas.

### Data collection and processing

**Figure 2:**
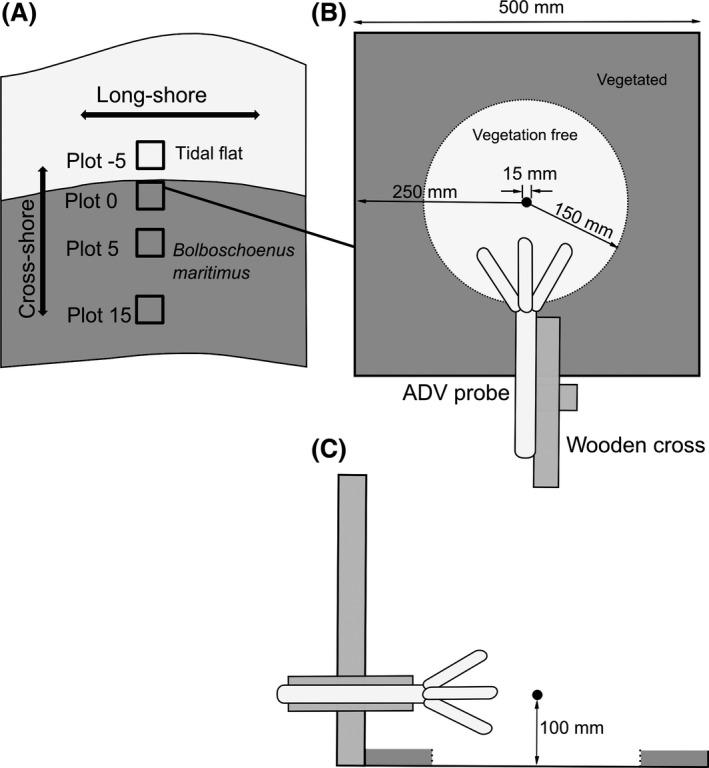
Scheme of the measurement locations. (A) Positioning of current measurements along the transect. (B) Top view of the installation design of ADV devices (black dot represents the measuring volume). (C) Side view of the installation design of ADV devices. Gray colouring indicates vegetated areas.

To evaluate how currents progress through the vegetation belt of *B. maritimus,* we took detailed measurements of long‐shore and cross‐shore velocities at one transect in study area A (Fig. 1) in April and August 2012 respectively. Measurements were conducted with four self‐recording Acoustic Doppler Velocimeters (ADV, Nortek Vector) at plots oriented on vegetation patterns: Plot 0 was placed at previous years’ marsh edge (Fig. 2A). All other plots were located at predefined distances from Plot 0 inside and in front of the *B. maritimus* belt (the latter is indicated by negative distance specifications).

Distances from the marsh edge were −5, 0, 5, and 15 m, corresponding to 1.19, 1.22, 1.26, and 1.31 m above sea level (Fig. 2A) and plot size was 0.5 **×** 0.5 m (Fig. 2B), the waterfront side of the plot being situated at the given distances. The ADVs were fixed horizontally on wooden crosses, positioned behind the plot in order to limit the effects of the support system on the measurements and to locate the measuring volume in the middle of the plot (Fig. 2A and B). Since the ADV is sensitive to objects between the probe and the sampling volume, the canopy was cut back within a 0.15 m radius around the sampling volume. The ADVs were positioned 0.1 m above the sediment in order to conduct simultaneous measurements for at least 1 h per flood at all plots (Leonard 1997). Particular caution was taken to minimize damage to vegetation during all operations. The instruments were programmed to measure up‐ and downstream (long‐shore) and on‐ and off‐shore (cross‐shore) velocity with a frequency of 4 Hz for up to 4 h, corresponding to the inundation time during one flood. In the course of preprocessing we excluded all measurements with missing data due to the dry falling of one of the ADVs, which resulted in time series of 60 min around high tide. Running means were calculated with a bin width of 1 min, corresponding to 240 measurements. To calculate flow velocity means for each location we averaged across the respective time series. Error bars on mean velocities represent mean ± 1 standard error of measured flow velocities from 60 min around high tide $\left(\sqrt{\text{var}(\text{flow velocity})/n\text{(flow velocity)}}\right)$. Changes during the growing season were quantified by comparing measurements without living vegetation (April) with measurements with maximal *B. maritimus* cover (August). To compare measurements in April with measurements in August, we used the plot in front of the vegetation (Plot −5) as reference. For this purpose, we calculated a normalized flow velocity by dividing the mean flow velocity in the vegetation plots (Plots 0, 5, 15) by the mean velocity in the vegetation‐free first plot (Plot −5). To quantify the effect of the living vegetation along the transect, we divided the results of the measurements in August by the results from April and fitted functions to the data. This was accomplished by least squares regression of the mean of the original or the logarithmized data.

To evaluate the effect of current velocity on plant morphology, we conducted further current measurements in front of 16 transects (Plot −5) in August 2013 (Fig. 1). The plots were again oriented on vegetation pattern and thus located at different elevations. For this purpose, we simultaneously measured at four transects during one flood, covering all 16 transects over the course of four floods. To gain a reference for comparing the different floods, we additionally measured during one more flood with one ADV at one of the transects of the four preceding measurements. In August 2013, we sampled three ramets of *B. maritimus* from the water front (Plot 0) and from 15 m inside the vegetation belt (Plot 15) at each transect (*n *=* *96) for biomechanic measurements. Mechanical resistance of these stems was assessed in a three point measuring setup with a universal testing machine (Zwick/Roell Modell BZ2.5/TN1S) (Vincent 1992; Kempe et al. 2013). The samples were loosely placed on the measuring device with two supporting points at 100 and 450 mm measured from the basal end of the stem. Flexural force was applied at 275 mm and the ramets then bent until breakage. Before measurement, all ramets were prepared as follows: the undermost leaves were removed from the stems and samples cut at 550 mm. Height and width of the plant cross‐section was measured at 100 mm and the mean used as a measure for stem diameter during further analyses. On the basis of the recorded flexural force, which was applied per bending distance, bending stiffness (*EI*) was calculated. For this purpose, the distance between the two supporting points (l = 350 mm) and the slope of the linear elastic range of the force deflection graph (*b*) was used: $\text{EI}=I^{3}x\left(b/48\right)$. As the minimum length‐diameter‐relation of a sample depends mainly on material properties of the sample (Lilholt 1886), l was determined experimentally (minimum length‐diameter‐ratio = 0.012 mm). Recorded data permitted the identification of the force, applied just before the buckling of the probe (*Fmax*).

In addition to these measurements, we recorded ramet density to characterize vegetation structure and evaluate the density effect on plant morphology. These measurements were conducted at all 16 transects at the marsh edge (Plot 0) and inside the vegetation belt (Plot 15). At each location, three plots of 0.25 m² were placed 5 m apart, parallel to the shoreline. The coordinates and elevation of all plots were recorded with a differential GPS. The plant density was measured at the peak of the growing season in 2012 (28 July – 02 August) and 2013 (30 July – 05 August). Data available from the Dryad Digital Repository: http://dx.doi.org/10.5061/dryad.np6b8.

The significance of differences in characteristics of plants from inside the vegetation and plants from the marsh edge was tested by the nonparametric Wilcoxon rank‐sum statistic because data were not normally distributed. The Wilcoxon rank‐sum statistic tests for differences between two groups on a single, ordinal variable without specific distribution (Wilcoxon 1945). Functions describing the correlation of stem diameter and flow velocity or plant stability were fitted to the data via linear regression using ordinary least squares for parameter estimation. To deal with heterogeneity of variance in the plant stability data, variables were log–transformed before analysis. All data analysis was carried out within the free software environment R 3.0.2 (R Development Core Team, 2014).

## Results

### Effect of *Bolboschoenus maritimus* vegetation on current velocity

**Figure 3:**
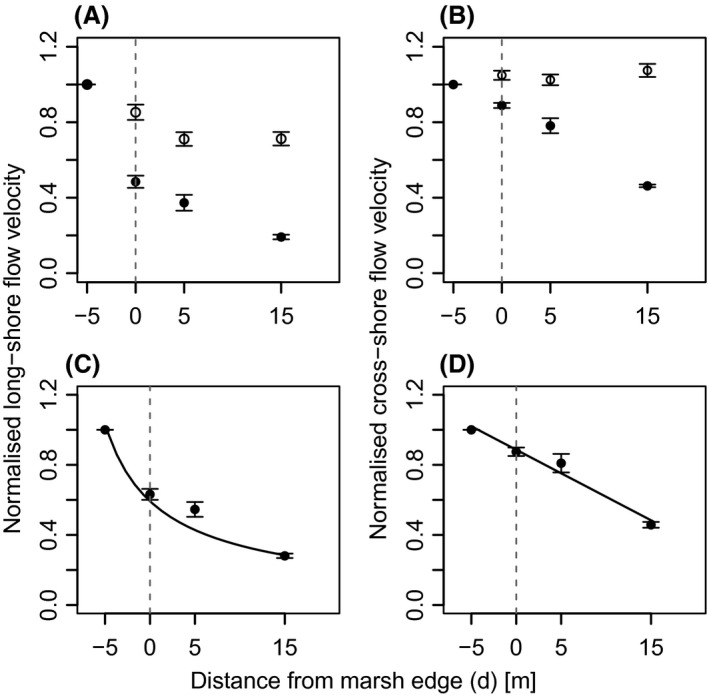
Normalized mean flow velocity at four distances from the marsh edge (*d*). The dashed line symbolizes the marsh edge. (A) and (B) Mean of measurements in April (white) and August (black) at the respective location. (C) and (D) Quantification of the effect of the vegetation on flow velocity (normalized flow velocity in August/normalized flow velocity in April). Continuous lines are the functions fitted to the data.

**Figure 4:**
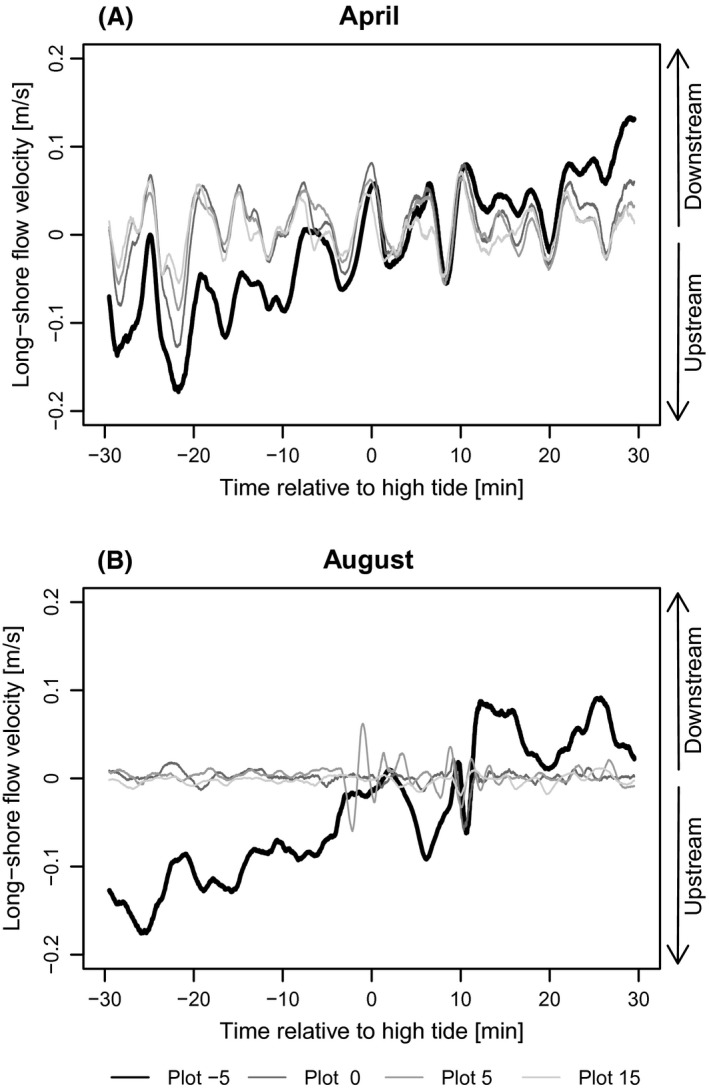
Running means of long‐shore flow velocity during one flood (30 min before until 30 min after high tide) in April (A) and August (B). Bin width for running mean: 1 min. Positive flow velocities represent downstream flow; negative velocities represent upstream flow respectively. Flow velocity in front of the vegetation is illustrated with a continuous black line. Vegetated plots are displayed in different shades of gray.

**Figure 5:**
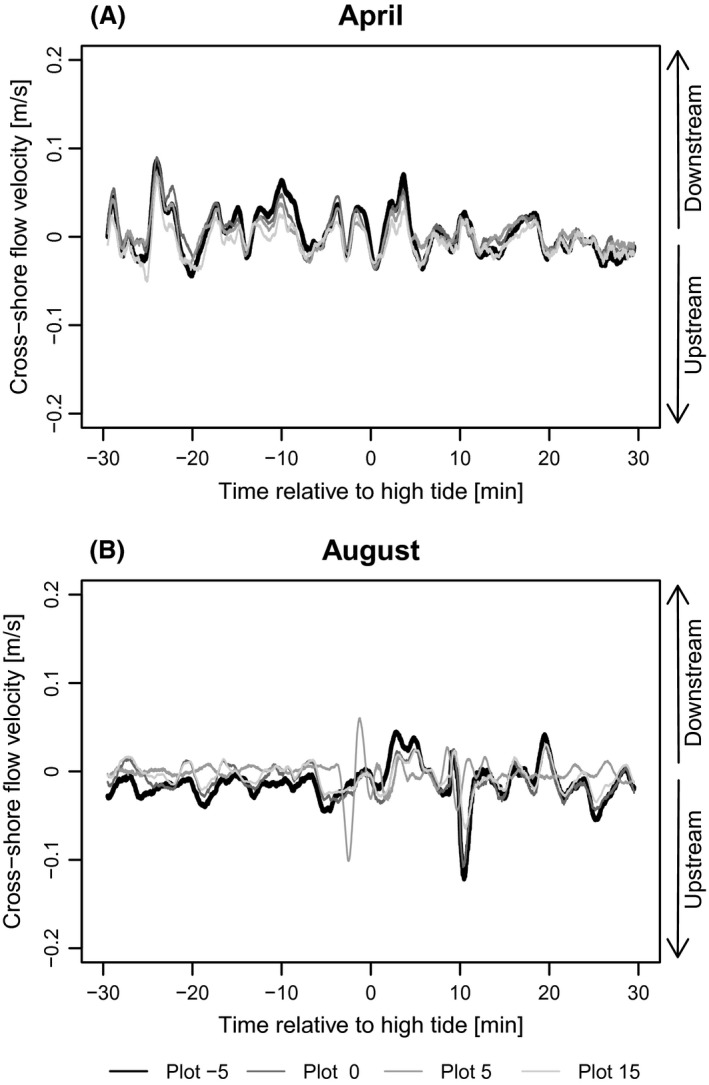
Running means of cross‐shore flow velocities during one flood (30 min before until 30 min after high tide) in April (A) and August (B). Bin width for running mean: 1 min. Positive flow velocities represent on‐shore flow; negative velocities represent off‐shore flow respectively. Flow velocity in front of the vegetation is illustrated with a continuous black line. Vegetated plots are displayed in different shades of gray.

Measured absolute long‐shore flow velocity at the current transect ranged from 0 to 0.18 m sec^−1^ with a mean of 0.03 m sec^−1^ and cross‐shore velocity ranged from 0 to 0.12 m sec^−1^ with a mean of 0.01 m sec^−1^. During the growing season flow velocity data revealed differences in the development of long‐shore and cross‐shore flow. Already in April, i.e. without living aboveground biomass, normalized long‐shore flow velocity $\text{flow velocity in plot}\;\left[{\text{msec}}^{-1}\right]/\text{flow velocity in the vegetation free first plot}\;\left[{\text{msec}}^{-1}\right]$ decreased with distance from the marsh edge (*d*) (Fig. 3A). However, this decrease was much stronger in August at the peak of the growing season, when mean flow velocities decreased by more than half immediately after entering the vegetation belt and then continued to gradually decrease with distance from the marsh edge. Normalized cross‐shore flow in April was very similar in all plots (Fig. 3B). In August, however, flow velocity decreased with distance from the mudflat into the vegetation. The net reduction of normalized flow velocity $\left(\frac{\text{normalized flow velocity in August}}{\text{normalized flow velocity in April}}\right)$, which we interpret as the buffering effect of living vegetation, was higher for long‐shore than for cross‐shore velocity (Fig. 3C and D). Owing to patterns in the data points, we fitted an exponential function to the data of normalized long‐shore velocity (Fig. 3C, $\text{normalized long‐shore velocity}=3.76\times {\left(d+10\right)}^{-0.8},\;P<0.001$, *R*² = 0.63) and a linear function for normalized cross‐shore velocity (Fig. 3D, normalized cross‐shore velocity=−0.027×d+0.89, *P* < 0.001, *R*² = 0.41). Running means of velocities around high tide show that the main long‐shore velocity changed from upstream to downstream after high tide (Fig. 4A). The comparison of long‐shore velocities in April and August (Fig. 4A and B) shows a reduced long‐shore velocity at the plots inside the vegetation belt in August. Although not as pronounced, cross‐shore velocity was as well‐damped by vegetation in August and showed lower amplitudes inside than in front of the vegetation belt, while no reduction could be observed in April (Fig. 5A and B).

### Morphological response of *Bolboschoenus maritimus* ramets

From the results of the current velocity measurements, we learned that plants inside the vegetation belt are less affected by currents than plants at the front. In analogy, the ramets of *B. maritimus* showed different growth types at the marsh edge and within the vegetation belt along all transects. Plants from the two study sites did not show any differences and were therefore regarded jointly in all further analyses.

**Figure 6:**
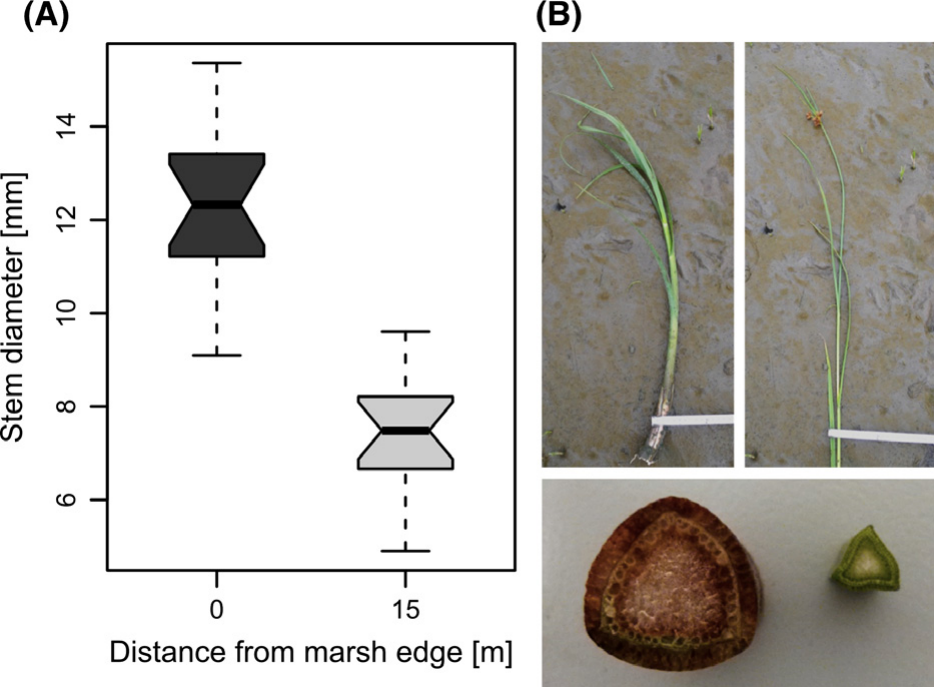
(A) Comparison of ramet diameters at two different distances from the marsh edge (B) Prominent examples of ramets (above) and cross sections (below) from the marsh edge (left) and from inside the vegetation belt (right).

The comparison of plants from the marsh edge (Plot 0) with plants from inside the vegetation (Plot 15) revealed a distinct difference in stem diameter: plants from Plot 15 were significantly thinner than plants from Plot 0 (Wilcoxon rank‐sum statistic: *P* < 0.001, *n *=* *96) (Fig. 6). Moreover, plants grew denser inside the vegetation belt (mean ramet density: 308 Stems m^−2^) than at the marsh edge (mean ramet density: 142 Stems m^−2^, Wilcoxon rank‐sum statistic: *P* < 0.001, *n *=* *96). However, considering plants from the marsh edge and from inside the vegetation belt separately, there was no effect of ramet density on stem diameter (Plot 0: *P* = 0.98, *n *=* *48, Plot 15: *P* = 0.34, *n *=* *48).

**Figure 7:**
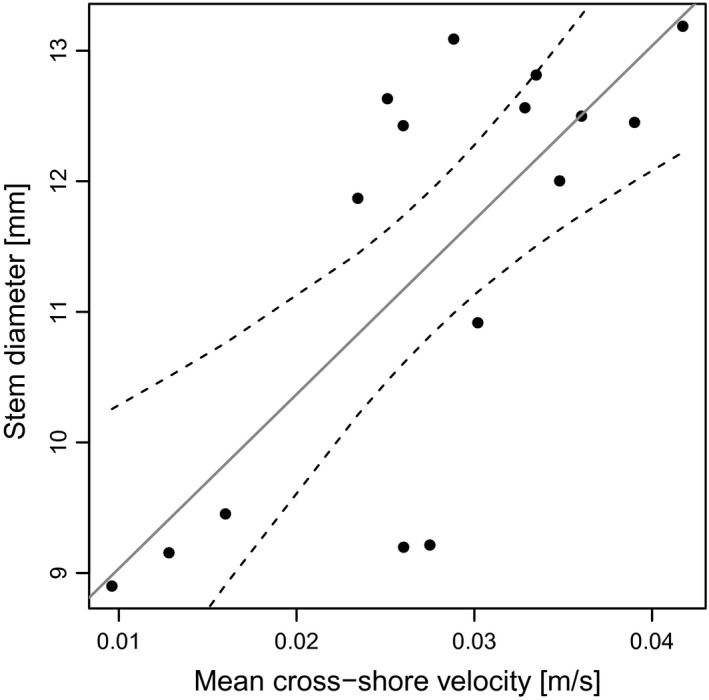
Correlation of mean cross‐shore flow velocity and stem diameter. Black dots represent the mean of measured values of stem diameter and flow velocity time series at each transect and the continuous line is the result of a linear regression. Dashed lines define the 95% confidence interval.

The current velocity measurements in front of all transects permitted the comparison of only plots from the marsh edge at the different sites. Mean long‐shore flow velocity at the sites ranged from 0.026 to 0.087 m sec^−1^ with a mean of 0.054 m sec^−1^ and mean cross‐shore velocity from 0.01 to 0.041 m sec^−1^ with a mean of 0.028 m sec^−1^. Current data from the two study areas did not show significant differences. We found that stem diameter was positively correlated with mean cross‐shore current velocity (stem diameter = 7.8 + 0.4 × mean cross‐shore current, *P* = 0.001, $R_{\mathrm{adj}}^{2}=0.52$, *n *=* *16) (Fig. 7) whereas stem diameter showed no correlation with mean long‐shore velocity or elevation.

**Figure 8:**
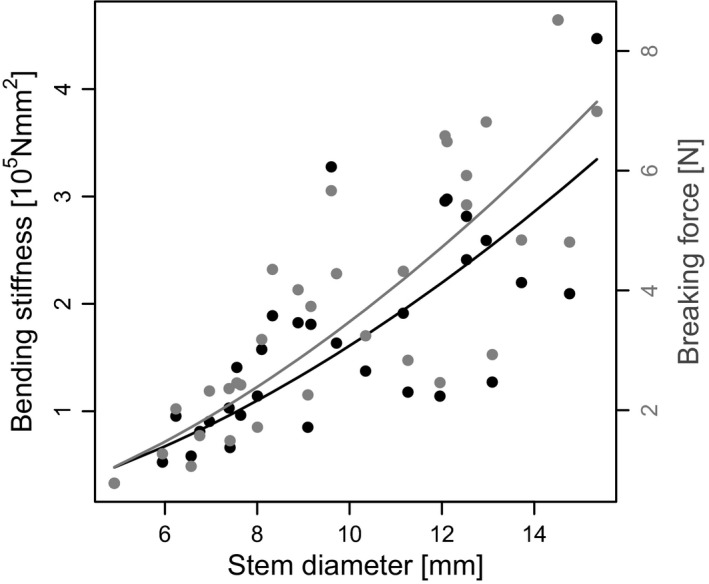
Correlation of stem diameter and breaking force and bending stiffness. Black and grey dots represent measured values and the respective lines are derived from linear regressions of the log‐transformed data.

**Figure 9:**
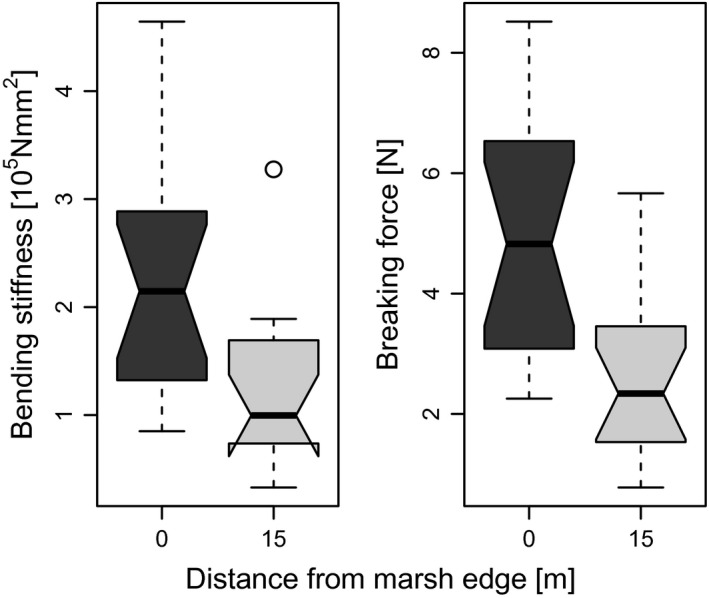
Bending stiffness and breaking force of ramets in relation to distance from marsh edge. Ramets from the marsh edge and from inside the vegetation differ significantly in bending stiffness and breaking force.

The biomechanical measurements – conducted to evaluate the adaptive value of higher stem diameter at locations with higher mechanical stress – showed a positive correlation of plant thickness and plant stability (bending stiffness = 0.03 × stem diameter^1.7^, *P* < 0.001, $R_{\mathrm{adj}}^{2}=0.67$, *n* = 32, breaking force = 0.07 × stem diameter^1.7^, *P* < 0.001, $R_{\mathrm{adj}}^{2}=0.7$, *n *=* *32) (Fig. 8). Consequently, plants from the front of the vegetation belt had a greater bending stiffness than plants from inside the vegetation (Wilcoxon rank‐sum statistic: *P* = 0.0019, *n *=* *16) and a significantly higher force had to be applied on them before breakage (Wilcoxon rank‐sum statistic: *P* < 0.001 *n *=* *16) (Fig. 9).

## Discussion

Previous work documented that flood risk is a growing concern for most coastal societies in the coming centuries (Hirabayashi and Kanae 2009). Temmerman et al. (2013) argued that the utilization of coastal ecosystems in flood protection could improve and support conventional coastal engineering solutions as it is more sustainable and cost effective with increasing flood risk. The ability of plants to adapt to environmental conditions, could further enhance the resistance of the whole vegetation belt against the mechanical stress of high current velocities.

In our study, we focused on the flow dampening potential of aboveground vegetation and its adaptation to the mechanic stress due to currents. On the one hand, we quantified the reduction of current velocity by *B. maritimus,* and on the other hand, we identified functional traits of *B. maritimus’* ramets which adapt to environmental differences and quantified their adaptive value.

### Effect of *Bolboschoenus maritimus* vegetation on current velocity

Although the April measurements represent a situation without living aboveground vegetation, they already identified a decrease in long‐shore current with distance from the marsh edge which could be the effect of decreasing water depth originating in the different elevation of the plots (see Methods – Data collection and processing). Cross‐shore velocity however, was stable along the entire transect (small variations lie within the range of natural variability).

Leonard and Luther (1995) discovered that mean flow velocity and flow energy inside vegetation are reduced by the plants’ dampening of large scale eddies. Our study confirmed a distinct difference between velocities in front of and inside the vegetation belt and a decrease of cross‐shore flow velocity with distance from the marsh edge into the marsh (Fig. 3).

Considering only the measurements in August (Fig. 3B), our results for cross‐shore currents are in accordance with Leonard and Croft (2006), who also found decreasing velocities with distance from the marsh edge, although with slightly higher dampening rates, for *Spartina alterniflora*. In contrary to the study of Leonard and Croft (2006) and others (e.g. Leonard and Luther 1995; Christiansen et al. 2000), our measurement design permitted the comparison of current velocity at the same site with and without living vegetation (Fig. 3A and B) and we considered cross‐shore currents additionally to long‐shore currents and were thus able to directly compare both (Figs. 3, 4, 5). Christiansen et al. (2000) found that flow velocity in vegetation is inversely related to distance from open water. Our results show that cross‐shore velocity decreases with distance from the marsh edge and that a 15 m wide belt of living *B. maritimus* vegetation is able to reduce an average cross‐shore current by more than 50% (Fig. 3D). As long‐shore flow runs parallel to the shore, it is not influenced by the vegetation growing normal to the marsh edge but by the vegetation stretch left and right along the shore (Bruno and Kennedy 2000). Thus the buffering vegetation stretch can be much larger than the distance to marsh edge, explaining the higher difference of long‐shore current between measurements in April and August (Fig. 3A and C). The reason for the lower vegetation effect on cross‐shore current (Fig. 3D), especially just behind the marsh edge, could consequently be that cross‐shore current is only buffered by the vegetation growing in direction of the tidal flat.

The results of this study confirm our hypothesis that for currents under normal, i.e. non‐stormy, conditions, the vegetation is able to buffer a large part of the current velocity. We quantified the reduction of long‐ and cross‐shore current velocity by *B. maritimus*. Under moderate conditions, this reduction can lead to higher sedimentation and lower erosion rates inside the vegetated marsh (Neumeier and Ciavola 2004) and thus promote natural surface accretion (Kirwan et al. 2010). However, we cannot extrapolate our results to storm surge conditions with much higher current velocities and water levels.

### Morphological response of *Bolboschoenus maritimus* ramets

The differences in ramet density at different locations within the vegetation belt could, as Charpentier and Stuefer (1999) already suggested, partly be an effect of the positioning of the ramets in the rhizome system: Ramets seem to grow less dense at the front of the belt, because here the belowground biomass is not as developed as in the older parts of the rhizome system lying further inside the vegetation belt.

Although different growth types of *B. maritimus* have been found in other studies (e.g. Lieffers and Shay 1981; Zákravský and Hroudová 1994), no study reported differences in stem diameter or attributed different growth forms to current velocity. With our results, we revealed that *B. maritimus* ramets exhibit different stem morphologies not only at different positions in the vegetation belt, but as well in the same position at sites with different current velocity (Fig. 7). In other studies, nutrient supply (Valiela et al. 1978) and elevation (Seliskar 1985) were found to effect stem diameter of marsh plants. We were able to exclude an effect of elevation, but have no information on nutrient supply, which could hence possibly contribute to the differences in plant morphology.

The simultaneous occurrence of thicker stems and lower ramet density at the marsh edge (Fig. 6) could give the impression that higher plant thickness was due to lower ramet density. By comparing *B. maritimus* diameters at different plots along the marsh edge, however, we found no correlation between ramet density and diameter, which could have been another plausible explanation for the two growth types.

For plants growing under such stressful conditions, maximum stability is an important functional trait to avoid breaking. In an experimental study on wave effects on *Phragmites australis*, Coops and Van der Velde (1996) did not find an effect on the morphology of this species. Other studies however, showed that plants can adapt to flow stress by morphological adjustments which either minimize mechanical forces or increase resistance to mechanical failure (Puijalon et al. 2005, 2008).

Our measurements revealed that plants growing at the front of the vegetation belt have significantly higher bending stiffness and a higher force has to be applied for breaking the ramets than inside the vegetation belt. The different growth forms could therewith provide an adaptive value in habitats with high mechanical stress (Figs. 8 and 9). With our study, we were able to show a positive correlation between plant thickness and cross‐shore current (Fig. 7) and we propose that the higher diameter of plants growing in the exposed front position could be a morphological adaptation to enhanced stress due to higher current velocity.

With the adjusted morphology of plants growing at the highly exposed marsh edge, the whole vegetation belt could be able to better resist the mechanical stress of high current velocities. As thicker plants are more stable and do not break as easily, this self‐adaptive effect thus increases the ability of *B. maritimus* to grow and persist in the pioneer zone and may hence contribute to ecosystem‐based coastal protection by reducing current velocity.

## Conflict of Interest

None declared.

## References

[ece31904-bib-0001] Alpert, P. , and E. L. Simms . 2002 The relative advantages of plasticity and fixity in different environments: when is it good for a plant to adjust? Evol. Ecol. 16:285–297.

[ece31904-bib-0002] Alpert, P. , and J. Stuefer . 1997 Division of labour in clonal plants Pp. 137–154 *in* de Kroon H. and van Groenendael J., eds. The ecology and evolution of clonal plants. Backhuys Publishers, Leiden.

[ece31904-bib-0003] Bal, K. D. , T. J. Bouma , K. Buis , E. Struyf , S. Jonas , H. Backx , et al. 2011 Trade‐off between drag reduction and light interception of macrophytes: comparing five aquatic plants with contrasting morphology. Funct. Ecol. 25:1197–1205.

[ece31904-bib-0004] Barrett, S. , C. Eckert , and B. Husband . 1993 Evolutionary processes in aquatic plant populations. Aquat. Bot. 44:105–145.

[ece31904-bib-0005] Boaden, P. J. S. , and R. Seed . 1988 An introduction to coastal ecology. Springer, Boston, MA, USA.

[ece31904-bib-0006] Bruno, J. F. 2000 Facilitation of cobble beach plant communities through habitat modification by *Spartina alterniflora* . Ecology 81:1179–1192.

[ece31904-bib-0007] Bruno, J. F. , and C. W. Kennedy . 2000 Patch‐size dependent habitat modification and facilitation on New England cobble beaches by Spartina alterniflora. Oecologia 122:98–108. 10.1007/PL00008841 28307962

[ece31904-bib-0008] Butcher, R. W. 1933 Studies on the ecology of rivers: I. On the distribution of macrophytic vegetation in the rivers of britain. J. Ecol. 21:58–91.

[ece31904-bib-0009] Charpentier, A. , and J. Stuefer . 1999 Functional specialization of ramets in *Scirpus maritimus* –splitting the tasks of sexual reproduction, vegetative growth, and resource storage. Plant Ecol. 141:129–136.

[ece31904-bib-0010] Christiansen, T. , P. L. Wiberg , and T. G. Milligan . 2000 Flow and sediment transport on a tidal salt marsh surface. Estuar. Coast. Shelf Sci. 50:315–331.

[ece31904-bib-0011] Clausen, J. , D. D. Keck , and W. M. Hisey . 1948 Experimental studies on the nature of species. III. Environresponses of climatic races of Achillea.

[ece31904-bib-0013] Clevering, O. A. , and M. P. J. Hundscheid . 1998 Plastic and non‐plastic variation in growth of newly established clones of Scirpus (Bolboschoenus) maritimus L. grown at different water depths. Aquat. Bot. 62:1–17.

[ece31904-bib-0014] Coops, H. , and G. Van der Velde . 1996 Effects of waves on helophyte stands: mechanical characteristics of stems of *Phragmites australis* and *Scirpus lacustris* . Aquat. Bot. 53:175–185.

[ece31904-bib-0015] Coops, H. , N. Geilen , and G. van der Velde . 1994 Distribution and growth of the helophyte species *Phragmites australis* and *Scirpus lacustris* in water depth gradients in relation to wave exposure. Aquat. Bot. 48:273–284.

[ece31904-bib-0016] Denny, M. 1988 Biology and the mechanics of the wave‐swept environment. Princeton, New Jersey, USA.

[ece31904-bib-0017] Dykyjová, D. 1986 Production ecology of *Bolboschoenus maritimus* (L.) Palla (*Scirpus maritimus* L. sl). Folia Geobot. Phytotax. 21:27–64.

[ece31904-bib-0018] Eriksson, O. , and L. Jerling . 1990 Hierarchical selection and risk spreading in clonal plants Pp. 79–94 *in Clonal growth in plants: regulation and function* eds van Groenendael J.M. and de Kroon H. SPB Academic Publishing, The Hague.

[ece31904-bib-0019] Gedan, K. B. , M. L. Kirwan , E. Wolanski , E. B. Barbier , and B. R. Silliman . 2010 The present and future role of coastal wetland vegetation in protecting shorelines: answering recent challenges to the paradigm. Clim. Change. 106:7–29.

[ece31904-bib-0020] Givnish, T. J. 2002 Ecological constraints on the evolution of plasticity in plants. Evol. Ecol. 16:213–242.

[ece31904-bib-0021] Hirabayashi, Y. , and S. Kanae . 2009 First estimate of the future global population at risk of flooding. Hydrol. Res. Lett. 3:6–9.

[ece31904-bib-0022] Houwing, E. J. 2000 Morphodynamic development of intertidal mudflats: consequences for the extension of the pioneer zone. Cont. Shelf Res. 20:1735–1748.

[ece31904-bib-0023] Hroudová, Z. , P. Zakrávský , M. Duchácek , and K. Marhold . 2007 Taxonomy, distribution and ecology of *Bolboschoenus* in Europe. Ann. Bot. Fenn. 44:81–102.

[ece31904-bib-0024] Karagatzides, J. D. , and I. Hutchinson . 1991 Intraspecific comparisons of biomass dynamics in *Scirpus americanus* and *Scirpus maritimus* on the Fraser River Delta. J. Ecol. 79:459–476.

[ece31904-bib-0025] van Katwijk, M. , and D. Hermus . 2000 Effects of water dynamics on *Zostera marina*: transplantation experiments in the intertidal Dutch Wadden Sea. Mar. Ecol. Prog. Ser. 208:107–118.

[ece31904-bib-0026] Kempe, A. , M. Sommer , and C. Neinhuis . 2013 A comparative analysis of the mechanical role of leaf sheaths of poaceae, juncaceae, and cyperaceae. J. Bot 2013:6.

[ece31904-bib-0027] Kirwan, M. L. , and J. P. Megonigal . 2013 Tidal wetland stability in the face of human impacts and sea‐level rise. Nature 504:53–60. 24305148 10.1038/nature12856

[ece31904-bib-0028] Kirwan, M. L. , G. R. Guntenspergen , A. D'Alpaos , J. T. Morris , S. M. Mudd , and S. Temmerman . 2010 Limits on the adaptability of coastal marshes to rising sea level. Geophys. Res. Lett., 37:23.

[ece31904-bib-0029] Le Hir, P. , W. Roberts , O. Cazaillet , M. Christie , P. Bassoullet , C. Bacher , et al. 2000 Characterization of intertidal flat hydrodynamics. Cont. Shelf Res. 20:1433–1459.

[ece31904-bib-0030] Leonard, L. A. 1997 Controls of sediment transport and deposition in an incised mainland marsh basin, southeastern North Carolina. Wetlands 17:263–274.

[ece31904-bib-0031] Leonard, L. A. , and A. L. Croft . 2006 The effect of standing biomass on flow velocity and turbulence in *Spartina alterniflora* canopies. Estuar. Coast. Shelf Sci. 69:325–336.

[ece31904-bib-0032] Leonard, L. A. , and M. E. Luther . 1995 Flow hydrodynamics in tidal marsh canopies. Limnol. Oceanogr. 40:1474–1484.

[ece31904-bib-0033] Lieffers, V. J. , and J. M. Shay . 1981 The effects of water level on the growth and reproduction of Scirpus *maritimus var. paludosus* . Can. J. Bot. 59:118–121.

[ece31904-bib-0034] Lieffers, V. J. , and J. M. Shay . 1982a Seasonal growth and standing crop of *Scirpus maritimus var. paludosus* in Saskatchewan. Can. J. Bot. 60:117–125.

[ece31904-bib-0035] Lieffers, V. J. , and J. M. Shay . 1982b Distribution and variation in growth of *Scirpus maritimus var. paludosus* on the Canadian prairies. Can. J. Bot. 60:1938–1949.

[ece31904-bib-0036] Lilholt, H. 1886 Mechanical Characterisation of Fibre Composite. Extract from Symposium on Mechanical Characterisation of fibre composite materials Pp. 129–146. *in* Pyrz R., ed. Mechanical Characterisation of Fibre Composite Materials. Aalborg University, Aalborg.

[ece31904-bib-0037] Lillebø, A. I. , M. A. Pardal , J. M. Neto , and J. C. Marques . 2003 Salinity as the major factor affecting *Scirpus maritimus* annual dynamics: evidence from field data and greenhouse experiment. Aquat. Bot. 77:111–120.

[ece31904-bib-0038] McAnally W. H., and Mehta A. J., eds. 2001 Coastal and estuarine fine sediment processes. Elsevier, Amsterdam.

[ece31904-bib-0039] Nepf, H. M. 1999 Drag, turbulence, and diffusion in flow through emergent vegetation. Water Resour. Res. 35:479–489.

[ece31904-bib-0040] Nepf, H. , and E. Vivoni . 2000 Flow structure in depth‐limited, vegetated flow. J. Geophys. Res. 105:28547–28557.

[ece31904-bib-0041] Neumeier, U. , and P. Ciavola . 2004 Flow resistance and associated sedimentary processes in a Spartina maritima salt‐marsh. J. Coastal Res. 20:435–447.

[ece31904-bib-0042] Puijalon, S. , and G. Bornette . 2004 Morphological variation of two taxonomically distant plant species along a natural flow velocity gradient. New Phytol. 163:651–660. 10.1111/j.1469-8137.2004.01135.x 33873737

[ece31904-bib-0043] Puijalon, S. , G. Bornette , and P. Sagnes . 2005 Adaptations to increasing hydraulic stress: morphology, hydrodynamics and fitness of two higher aquatic plant species. J. Exp. Bot. 56:777–786. 15642713 10.1093/jxb/eri063

[ece31904-bib-0044] Puijalon, S. , J.‐P. Léna , N. Rivière , J.‐Y. Champagne , J.‐C. Rostan , and G. Bornette . 2008 Phenotypic plasticity in response to mechanical stress: hydrodynamic performance and fitness of four aquatic plant species. New Phytol. 177:907–917. 18275493 10.1111/j.1469-8137.2007.02314.x

[ece31904-bib-0045] Puijalon, S. , T. J. Bouma , C. J. Douady , J. van Groenendael , N. P. R. Anten , E. Martel , et al. 2011 Plant resistance to mechanical stress: evidence of an avoidance‐tolerance trade‐off. New Phytol. 191:1141–1149. 21585390 10.1111/j.1469-8137.2011.03763.x

[ece31904-bib-3000] R Core Team (2013). R: A language and environment for statistical computing. R Foundation for Statistical Computing, Vienna, Austria. URL http://www.R-project.org/

[ece31904-bib-0046] Richards, C. L. , S. C. Pennings , and L. A. Donovan . 2005 Habitat range and phenotypic variation in salt marsh plants. Plant Ecol. 176:263–273.

[ece31904-bib-0047] Seliskar, D. 1985 Morphometric variations of five tidal marsh halophytes along environmental gradients. Am. J. Bot. 72:1340–1352.

[ece31904-bib-0048] Shepard, C. C. , C. M. Crain , and M. W. Beck . 2011 The protective role of coastal marshes: a systematic review and meta‐analysis. PLoS ONE 6:e27374. 22132099 10.1371/journal.pone.0027374 PMC3223169

[ece31904-bib-0049] Suzuki, J. I. , and J. F. Stuefer . 1999 On the ecological and evolutionary significance of storage in clonal plants. Plant Spec. Biol. 14:11–17.

[ece31904-bib-0050] Szmeja, J. , and A. Galka . 2008 Phenotypic responses to water flow and wave exposure in aquatic plants. Acta Soc. Bot. Pol. 77:59–65.

[ece31904-bib-0051] Temmerman, S. , T. J. Bouma , G. Govers , Z. B. Wang , M. B. De Vries , and P. M. J. Herman . 2005 Impact of vegetation on flow routing and sedimentation patterns: three dimensional modeling for a tidal marsh. J. Geophys. Res. 110:F04019.

[ece31904-bib-0052] Temmerman, S. , P. Meire , T. J. Bouma , P. M. J. Herman , T. Ysebaert , and H. J. De Vriend . 2013 Ecosystem‐based coastal defence in the face of global change. Nature 504:79–83. 24305151 10.1038/nature12859

[ece31904-bib-0053] Valiela, I. , J. M. Teal , and W. G. Deuser . 1978 The nature of growth forms in the salt marsh grass *Spartina alterniflora* . Am. Nat. 112:461.

[ece31904-bib-0054] Vincent, J. F. V. 1992 Biomechanics – Materials, A Practical Approach Pp 42–43. *in* Vincent J. F. V., ed. Biomechanics – Materials, a practical approach. Oxford University Press, Oxford.

[ece31904-bib-0055] Vogel, S. 1994 Life in moving fluids: the physical biology of flow. Princeton University Press, Princeton.

[ece31904-bib-0056] Wilcoxon, F. 1945 Individual comparisons by ranking methods. Biometr. Bull. 1:80–83.

[ece31904-bib-0057] Zákravský, P. , and Z. Hroudová . 1994 The effect of submergence on tuber production and dormancy in two subspecies of *Bolboschoenus maritimus* . Folia Geobot. 29:217–226.

